# Generative Artificial Intelligence (AI)–Assisted Self-Diagnosis and the Patient-Physician Relationship: Mixed Methods Study of Calibration, Participation, and Trust

**DOI:** 10.2196/95072

**Published:** 2026-08-18

**Authors:** Wuyao Ding, Junxiu Wang

**Affiliations:** 1 School of Mental Health, Wenzhou Medical University, University Town, Chashan, Ouhai District, Wenzhou, Zhejiang, 325035, China, +86 18518969163; 2 School of Media and Communication, Shanghai Jiao Tong University, Shanghai, China

**Keywords:** generative artificial intelligence, self-diagnosis, patient-physician relationship, illness appraisal, shared decision-making, trust in physician, mixed methods research, health communication

## Abstract

**Background:**

Patients increasingly use generative AI to interpret symptoms and seek health information, yet limited evidence shows how AI-assisted self-diagnosis is integrated into care-seeking and related to clinical interactions and patient–physician relationships.

**Objective:**

This study examined how AI-assisted self-diagnosis is incorporated into care-seeking processes and how it relates to patient participation in clinical encounters and trust in physicians.

**Methods:**

An exploratory sequential mixed methods design was used. In the qualitative phase, Chinese adults who had used generative AI to interpret symptoms, appraise possible conditions, or seek health advice within the previous year were purposively recruited through Xiaohongshu, WeChat Moments, and WeChat groups. Semistructured interviews were conducted from August 20, 2025, to January 10, 2026, and analyzed using reflexive thematic analysis. In the quantitative phase, an anonymous web-based survey was conducted in China through Huixiang Data from January 25, 2026, through January 28, 2026. Adults who had used generative AI for health consultation involving symptom interpretation or preliminary self-diagnosis within the previous 6 months were recruited through convenience sampling. Measures included perceived AI-assisted self-diagnosis quality, calibrated illness appraisal, patient participation, diagnosis validation, diagnosis comprehension, trust in physicians, AI use frequency, trust in health information sources, and demographic characteristics. Trust in physicians was assessed using 5 adapted items covering competence, integrity, and benevolence. Descriptive statistics, Pearson correlations, and PROCESS mediation analyses were performed.

**Results:**

Qualitative findings (n=48) indicated that AI-assisted self-diagnosis was commonly used in a prediagnostic gray zone for preliminary orientation, informal triage, and interim self-management. Participants described AI as helping them appraise illness severity, prepare for consultations, ask questions, and understand physicians’ diagnoses and reasoning. Quantitative findings (n=546) were consistent with these patterns. Perceived AI quality was positively associated with calibrated illness appraisal (*b*=0.57, 95% CI 0.49-0.64), which was positively associated with patient participation (*b*=0.35, 95% CI 0.28-0.43). The indirect association was significant (estimate=0.20, 95% bootstrap CI 0.14-0.26). Perceived AI quality was also associated with diagnosis validation (*b*=0.69, 95% CI 0.61-0.76) and diagnosis comprehension (*b*=0.65, 95% CI 0.58-0.73), which were associated with trust in physicians (*b*=0.15, 95% CI 0.07-0.23 and *b*=0.20, 95% CI 0.12-0.28, respectively). The corresponding indirect associations were 0.10 (95% bootstrap CI 0.04-0.17) through diagnosis validation and 0.13 (95% bootstrap CI 0.07-0.19) through diagnosis comprehension.

**Conclusions:**

Generative AI may function as an informational intermediary across the care-seeking process rather than undermine medical authority. By supporting illness appraisal, consultation preparation, and postconsultation understanding, AI-assisted self-diagnosis may be associated with greater patient participation and trust in physicians.

## Introduction

### Background

Generative artificial intelligence (AI) refers to systems capable of producing novel content, including text, images, audio, video, or other media, in response to user inputs [1]. In health care, generative AI is increasingly used to answer health-related questions, interpret symptoms, explore possible causes, and help users make preliminary judgments about whether professional care is warranted [2,3]. This use of AI to assess one’s own health condition without direct guidance from a health care professional is referred to as AI-assisted self-diagnosis [3-5].

Despite the growing use of AI-assisted self-diagnosis, existing research has focused primarily on users’ adoption motives, evaluations of the accuracy of AI-generated health information, and intentions to continue using these tools [4-6]. Much less is known about how AI-assisted self-diagnosis is incorporated into real-world care-seeking trajectories and clinical encounters or how it relates to patients’ interactions with physicians. This gap motivated this mixed methods study.

### Self-Diagnosis: From the Internet Era to the AI Era

Internet-based self-diagnosis offers a useful baseline. Search engines, health websites, and online forums have long enabled patients to access medical information before consultations, sometimes improving health literacy and encouraging more active participation [7-9]. At the same time, online health information is often fragmented, inconsistent, and difficult for laypersons to evaluate [8,10,11]. Search results can foreground severe or unlikely conditions and intensify health anxiety or cyberchondria [12-14]. These limits help explain why patients often continue to treat physicians as the primary authority in medical decision-making even when they seek information online [8,10].

Generative AI changes this environment by altering how health information is presented and explored. Unlike conventional web searches, conversational AI can synthesize information into coherent responses, support iterative questioning, and tailor explanations to user-provided details [1,15]. Studies examining user experiences with generative AI in health contexts have shown that individuals value its accessibility, conversational interaction, and ability to provide synthesized and personalized explanations [15]. Users also perceive these features as helpful for acquiring health-related knowledge, improving their understanding of symptoms and possible causes, and making sense of uncertain health information [2,15].

Despite these perceived advantages, a systematic review comparing online symptom assessment applications, large language models (LLMs), and laypeople found that LLM self-triage accuracy remained moderate. It concluded that such systems should neither be universally recommended nor discouraged but evaluated according to specific use cases and user groups [16]. The review also identified a broader limitation of accuracy-oriented research: Technological systems and laypeople have generally been evaluated separately, leaving limited evidence on how users interpret and incorporate technological recommendations into actual self-triage decisions.

Reflecting this shift from isolated performance evaluation to situated human-technology interaction, a technology-supported self-triage decision-making model conceptualizes self-triage as a joint human-technology process [17]. Technological systems support information gathering and analysis, while users integrate system outputs with bodily experiences, prior knowledge, emotional responses, practical constraints, and, where available, professional medical advice before deciding whether and how to seek care.

From this human-in-the-loop perspective, AI-assisted illness appraisal is not merely a technological output but an interpretive process through which users relate AI-generated information to their symptoms and circumstances. Accordingly, the first aim of this study was to examine how users incorporate AI-generated health information into illness appraisal, including how they interpret symptom severity, manage uncertainty, and make preliminary judgments about the need for professional care.

### From AI-Assisted Self-Diagnosis to Clinical Interaction

Beyond illness appraisal, AI-assisted self-diagnosis may matter because AI-generated information can be carried into subsequent clinical encounters. Two established strands of research on patient-physician communication and relationships are particularly relevant in this context. One emphasizes patient participation in clinical encounters [8,18-20]. The other focuses on patients’ trust in physicians [21-24].

Patient participation refers to patients’ active communicative and deliberative involvement in medical consultations, including describing symptoms, asking questions, expressing concerns, understanding physicians’ explanations, participating in discussions about diagnosis or treatment options, and actively sharing in the decision-making process [18-20]. Prior research on online health information-【seeking has shown that patients who obtain health information before visiting physicians may enter consultations with greater preparedness and confidence [8]. Generative AI may further strengthen this preparatory function by synthesizing users’ symptom information, clarifying medical terminology, and supporting iterative questioning. Building on this possibility, the second aim of the study was to examine how AI-assisted symptom interpretation relates to consultation preparation and patient participation in medical encounters.

Beyond its potential association with patient participation, AI-assisted self-diagnosis may also be associated with trust in physicians. Trust in physicians refers to patients’ confidence in physicians’ professional competence, integrity (or honesty), and benevolence (or fidelity) under conditions of medical uncertainty and vulnerability [21-23]. Trust in physicians is a foundational component of effective health care, facilitating patient disclosure, adherence to medical recommendations, and cooperative decision-making [24,25]. Trust also stabilizes clinical uncertainty by enabling patients to accept diagnostic judgments and treatment plans despite informational asymmetry [21,22].

The emergence of AI-based health consultation tools has prompted debate regarding whether this authority structure may be reshaped. Some scholars suggest that AI systems may increasingly function as alternative sources of medical knowledge, which may enable patients to critically evaluate or question physicians’ recommendations [26-28], potentially reducing their trust in physicians. Others argue that digital health technologies are more likely to function as supportive informational tools that complement rather than replace professional expertise [29]. Against this background, the third aim of the study was to examine how AI-generated explanations relate to diagnosis validation, diagnosis comprehension, and trust in physicians.

## Methods

### Study Design

This study examined how generative AI-assisted self-diagnosis is incorporated into patients’ care-seeking processes and how it relates to participation in clinical encounters and trust in physicians. An exploratory sequential mixed methods design was used [30]. In the qualitative phase, semistructured interviews were conducted with individuals who had used generative AI tools for health consultation to explore how AI-assisted symptom interpretation was experienced and integrated into care-seeking trajectories. Findings from the qualitative analysis were then operationalized and examined in the quantitative phase through a web-based survey. By combining qualitative exploration with quantitative examination, the study offers a process-oriented account of how generative AI is embedded in contemporary health information practices and how its use may shape patient-physician interactions.

### Qualitative Phase

#### Research Questions

An interpretive qualitative approach was used to examine how individuals integrate AI-based health consultation tools into everyday medical decision-making. The analysis was guided by 4 research questions (RQs):

- RQ1: In what contexts and for what reasons do patients use generative AI for self-diagnosis?
- RQ2: How is generative AI-assisted self-diagnosis related to patients’ illness-response orientations, including emotional consequences and decisions to seek professional care after symptom appraisal?
- RQ3: How are experiences with generative AI-assisted self-diagnosis carried into clinical encounters and reflected in patient-physician communication?
- RQ4: How is generative AI use for self-diagnosis related to patients’ trust in physicians and perceptions of professional authority?

#### Participants and Sampling

Participants were recruited through open calls then selected using purposive maximum-variation sampling. Eligible participants were adults aged 18 years or older who had used generative AI within the previous year and could recall and describe a specific generative AI use episode involving symptom interpretation, preliminary condition appraisal, or health consultation.

Recruitment notices were posted on Xiaohongshu, WeChat Moments, and WeChat groups. They described the study as an interview study of AI-assisted health consultation and invited individuals with relevant experience to contact the research team. Interested individuals completed a brief eligibility and background screening before interviews were scheduled.

Eligible volunteers were purposively selected to capture variation in demographic characteristics and AI-assisted health consultation experiences. Selection considered age, gender, AI use frequency, types of generative AI tools used, health concerns discussed with AI, and whether AI consultation was followed by professional medical care. Recruitment and selection were reviewed iteratively during data collection. When an experiential or demographic category was underrepresented, subsequent recruitment targeted individuals with those characteristics. Recruitment continued until thematic saturation, defined as the point at which additional interviews produced no substantively new thematic categories or conceptual dimensions. In total, 48 participants were interviewed; detailed characteristics are reported in the Results section.

#### Data Collection

Semistructured interviews were conducted between August 20, 2025, and January 10, 2026. Most were 1-on-1 online voice calls via WeChat or Tencent Meeting, with a small number conducted in person. Interviews lasted approximately 30 minutes to 45 minutes and were conducted in Mandarin Chinese.

An interview guide ensured thematic coherence while allowing in-depth exploration. Core domains included contexts and motivations for AI use, comparative trust in AI and physicians, the perceived role of AI in decisions to seek professional care, use of AI-generated information in clinical encounters, responses to discrepancies between AI and physician advice, and perceived changes in the overall care experience. Participants were encouraged to recount specific clinical encounters to provide contextually grounded narratives. Two pilot interviews were used to refine the guide for clarity and natural flow (Table S1, Multimedia Appendix 1).

With participants’ consent, all interviews were audio-recorded, transcribed verbatim in Chinese, and anonymized before analysis. Because analysis was conducted on the original Chinese transcripts rather than translated materials, back-translation was not used. Illustrative quotations were translated into English for reporting and checked against the Chinese transcripts to preserve semantic accuracy.

#### Qualitative Data Analysis

Interview transcripts were analyzed using reflexive thematic analysis following a 6-phase framework [31,32]. Data collection and analysis proceeded concurrently and iteratively. Analysis began following transcription of the first interview and continued throughout the data collection period. As each transcript became available, it was read repeatedly; coded; and compared with earlier transcripts to build familiarity, identify preliminary patterns, and progressively refine emerging interpretations. Initial coding captured meaningful segments related to AI use, illness appraisal and response, clinical interaction, and trust. Codes were then grouped into candidate themes based on conceptual coherence and interpretive relevance. These themes were reviewed against coded extracts and the full dataset to assess internal coherence, empirical grounding, and distinctiveness and were subsequently refined, named, and organized into a thematic structure.

Consistent with reflexive thematic analysis, rigor was pursued through iterative engagement with the data, reflexive memoing, attention to discrepant cases, and critical review of interpretations rather than intercoder reliability testing. The first author conducted all coding and developed the initial thematic structure. Her training in psychology, health communication, and AI-related health research sensitized the analysis to trust, patient participation, medical authority, and digital health information practices.

The second author then reviewed the thematic framework and supporting interview evidence. As an established scholar in social psychology and medical sociology with extensive experience in health behavior and social processes in health care, the second author contributed an additional disciplinary perspective. The review examined the clarity of theme definitions, alignment between thematic claims and supporting data, and overall analytic coherence.

To limit overinterpretation arising from the researchers’ prior interests, the analysis prioritized participants’ concrete accounts of AI use and clinical encounters; attended to negative and discrepant cases; and used analytic memos to document coding decisions, emerging conceptual links, uncertainties, and revisions. The final thematic structure is presented in the Results section.

### Quantitative Phase

#### Hypotheses

The quantitative phase examined whether the experiential mechanisms identified qualitatively were also evident among individuals who had used generative AI for health consultation. Based on the qualitative findings, we proposed the following hypotheses (H):

- H1: Perceived quality of AI-assisted self-diagnosis is positively associated with calibrated illness appraisal.
- H2: Calibrated illness appraisal is positively associated with patient participation in clinical encounters.
- H3: Perceived quality of AI-assisted self-diagnosis has a statistically significant positive indirect association with patient participation in clinical encounters through calibrated illness appraisal.
- H4: Perceived quality of AI-assisted self-diagnosis is positively associated with diagnosis validation.
- H5: Perceived quality of AI-assisted self-diagnosis is positively associated with diagnosis comprehension.
- H6: Diagnosis validation is positively associated with trust in physicians.
- H7: Diagnosis comprehension is positively associated with trust in physicians.
- H8: Perceived quality of AI-assisted self-diagnosis has statistically significant positive indirect associations with trust in physicians through diagnosis validation and diagnosis comprehension.

A cross-sectional, anonymous, web-based survey was conducted in China from January 25, 2026, through January 28, 2026, through Huixiang Data, a Chinese online survey panel. The data collection period was predetermined, and all respondents who met the eligibility and data quality criteria and completed the survey during this period were included in the analytic sample.

Participants were recruited through convenience sampling via Huixiang Data’s online recruitment system. Recruitment materials described the study as a survey of experiences with generative AI–assisted health consultation and subsequent interactions with health care professionals. Participation was voluntary and anonymous.

Eligible respondents were aged 18 years or older and, within the previous 6 months, had both used generative AI for health-related consultation involving symptom interpretation or preliminary self-diagnosis and attended at least one medical consultation with a physician or other health care professional. These criteria ensured that respondents had relevant experience with both AI-generated health information and clinical encounters. The AI consultation and medical visit were not required to concern the same health episode, because the survey assessed respondents’ overall experiences rather than following a single AI-to-care episode longitudinally.

Eligibility was assessed at the beginning of the survey and verified again during data screening. An embedded attention check item was used to identify inattentive responses. Of the 560 initial responses, 5 were excluded for failing the attention check, and 9 were excluded because the respondents were younger than 18 years, yielding a final analytic sample of 546. All responses were also screened for completeness. No identifiable personal information nor medical record information was collected.

The final sample was considered adequate for the planned regression-based mediation analyses. Fritz and MacKinnon simulation results indicate that mediation analyses using bias-corrected bootstrap confidence intervals generally require approximately 400‐460 participants to detect small-to-medium indirect associations under common combinations of a and b path magnitudes [33]. The final sample of 546 therefore exceeded these recommendations.

#### Measures

The questionnaire was developed through an integrative operationalization process combining qualitative themes with established measures from prior research. Unless otherwise specified, all multi-item constructs used 5-point Likert-type scales ranging from 1 (strongly disagree) to 5 (strongly agree). Negatively worded items were reverse-coded before scale construction. Scale scores were calculated by averaging item responses, with higher scores indicating higher levels of each construct. Researchers with expertise in health communication and medical AI reviewed newly developed or adapted items for conceptual clarity and content validity. The survey was pilot-tested with 30 respondents to assess item clarity, questionnaire flow, and completion time, and minor wording revisions were made based on feedback. Complete items are provided in Table S2 Multimedia Appendix 1.

*Perceived quality of AI-assisted self-diagnosis* refers to evaluations of the clarity, relevance, accuracy, and trustworthiness of AI-generated explanations used to interpret symptoms and consider possible health conditions. It was measured using 7 items (Cronbach *α*=0.92) adapted from information quality scales in technology adoption or information systems research [34] and modified to reflect the conversational and synthesized nature of generative AI outputs.

*Calibrated illness appraisal* refers to respondents’ perceived ability to interpret symptoms proportionately and regulate emotional responses after AI-assisted self-diagnosis. It was treated as an empirically derived construct from the qualitative phase rather than a pre-existing standardized measure. Consisting of 5 items (Cronbach *α*=0.88), this construct assessed whether AI supported more reasonable judgments of symptom severity, clearer understanding of possible causes, reduced anxiety about minor symptoms, emotional composure, and timely recognition of conditions requiring professional care. The items were developed inductively from themes concerning proportionate symptom interpretation, uncertainty regulation, and care-seeking judgment and informed by prior research on symptom checkers and self-diagnosis [4,35].

*Patient participation in clinical encounters* refers to active communicative and deliberative involvement during medical consultations after AI-assisted preparation. Consisting of 4 items (Cronbach *α*=0.82), this construct assessed active engagement. The items were developed based on interview themes concerning symptom narration, question asking, and deliberative involvement, with reference to the broader conceptual literature on shared decision-making and patient engagement [36,37].

Diagnosis validation and diagnosis comprehension were derived from the qualitative interviews. *Diagnosis validation* refers to perceived alignment between AI-generated assessments and physicians’ diagnoses or treatment recommendations and was measured using 3 items (Cronbach *α*=0.85). *Diagnosis comprehension* refers to the extent to which AI-assisted explanations improved understanding of physicians’ diagnostic reasoning and treatment decisions and was measured using 3 items (Cronbach *α*=0.84).

*Trust in physicians* refers to confidence in physicians’ professional competence, integrity, and benevolence. This relational trust construct was measured using 5 items (Cronbach *α*=0.85) adapted from established physician-trust scales [21,24,38].

The survey also measured AI use frequency and trust in multiple health information sources for contextual and comparative purposes. AI-assisted self-diagnosis frequency was assessed through a subjective frequency rating from very infrequent to very frequent. Trust in each health information source was measured with a separate single-item indicator adapted from the Health Information National Trends Survey [39]. Respondents separately rated trust in physicians as a health information source, family or friends, traditional media (newspapers or magazines, television, and radio), online news platforms, government health agencies, social media platforms, search engines, blogs, and AI chatbots. These contextual source-trust items were distinct from the 5-item relational trust scale used as the primary outcome.

Demographic information included gender, age, education, marital status, employment status, residential location, income stability, health care–related certification/qualification, and chronic disease status.

### Statistical Analysis

All analyses were conducted in SPSS version 24 (IBM Corp), with mediation analyses performed using the PROCESS macro (version 4.2; Andrew F Hayes). Descriptive statistics summarized demographic characteristics, AI use patterns, and key study variables. Internal consistency of multi-item scales was assessed using Cronbach α, and Pearson correlations examined bivariate associations among the main constructs.

Regression-based mediation analyses tested 2 prespecified models derived from the mixed methods framework. In the engagement model, perceived quality of AI-assisted self-diagnosis was specified as the independent variable, calibrated illness appraisal as the mediator, and patient participation in clinical encounters as the outcome. In the authority-related model, perceived quality of AI-assisted self-diagnosis was specified as the independent variable, diagnosis validation and diagnosis comprehension as parallel mediators, and trust in physicians as the outcome. No covariates were included since the models were intended to examine theoretically specified relationships among the focal constructs rather than covariate-adjusted effects. Given the cross-sectional design, the findings were interpreted as indirect associations rather than evidence of causal mediation.

Indirect associations were estimated using 5000 bootstrap resamples and considered statistically significant when the 95% bootstrap CI excluded zero. We report unstandardized coefficients, standard errors, *t* values, *P* values, 95% CIs, *R*^2^ values, *F* statistics, and bootstrapped indirect effect estimates. Because PROCESS uses regression-based models rather than structural equation modeling, global fit indices such as comparative fit index (CFI), Tucker-Lewis index (TLI), root mean square error of approximation (RMSEA), and standardized root mean squared residual (SRMR) were not applicable.

### Ethical Considerations

The study protocol was approved by the Ethics Committee of Wenzhou Medical University (number 2026041) and the Ethics Committee of the First Affiliated Hospital of Wenzhou Medical University (number 2025-KY-187).

## Results

### Participants

The demographic characteristics for the 48 individuals who participated in the interviews are summarized in Table 1. The survey analysis included 546 respondents, with detailed sample characteristics provided in Table 2.

**Table 1.** Interview participants’ characteristics (n=48).

Characteristic	Results, n (%)^a^
Gender	
Male	24 (50)
Female	24 (50)
Age group (years)	
18‐25	32 (66.7)
26‐30	11 (22.9)
31‐40	4 (8.3)
51‐60	1 (2.1)
Highest education level	
Graduate or higher	18 (37.5)
Undergraduate/bachelor	20 (41.7)
Junior college/associate degree	7 (14.6)
High school/vocational	2 (4.2)
Junior high school or below	1 (2.1)
Occupation	
Student	18 (37.5)
Employed/in service	23 (47.9)
Freelance/self-employed	4 (8.3)
Other (including caregiver/parent)	2 (4.2)
Retired	1 (2.1)
Residential setting	
Urban/city	39 (81.3)
Township	7 (14.6)
Rural/village	2 (4.2)

a Due to rounding, percentages might not total 100% for each characteristic.

**Table 2.** Survey participants’ characteristics (n=546).

Characteristic	Results, n (%)^a^
Gender	
Male	242 (44.3)
Female	304 (55.7)
Age group (years)	
18‐25	87 (15.9)
26‐30	113 (20.7)
31‐35	116 (21.2)
36‐40	69 (12.6)
41‐45	72 (13.2)
46‐50	63 (11.5)
51‐55	6 (1.1)
56‐60	9 (1.6)
>60	11 (2)
Education level	
Middle school or below	15 (2.7)
High school or vocational school	16 (2.9)
Associate degree	117 (21.4)
Bachelor’s degree	330 (60.4)
Master’s degree	59 (10.8)
Doctoral degree	5 (0.9)
Other	4 (0.7)
Marital status	
Married	385 (70.5)
Partner relationship	53 (9.7)
Single	96 (17.6)
Widowed	2 (0.4)
Divorced	5 (0.9)
Other	5 (0.9)
Employment status	
Full-time employment	361 (66.1)
Self-employed	65 (11.9)
Part-time employment	42 (7.7)
Retired	9 (1.6)
Disability benefits recipient	1 (0.2)
Full-time student	47 (8.6)
Unemployed, seeking work	7 (1.3)
Unemployed, not seeking work	11 (2)
Homemaker	1 (0.2)
Other	2 (0.4)
Income stability	
Yes	487 (89.2)
No	59 (10.8)
Health care–related certification/qualification	
Yes	63 (11.5)
No	483 (88.5)
Residence	
Urban	412 (75.5)
Rural	134 (24.5)
Chronic diseases requiring regular monitoring or treatment	
Yes	358 (65.6)
No	188 (34.4)
AI^b^-assisted self-diagnosis frequency	
1, very infrequent	13 (2.4)
2	80 (14.7)
3	151 (27.7)
4	213 (39)
5, very frequent	89 (16.3)

a Due to rounding, percentages might not total 100% for each characteristic.

b AI: artificial intelligence.

### Interview Results

**Figure 1.**
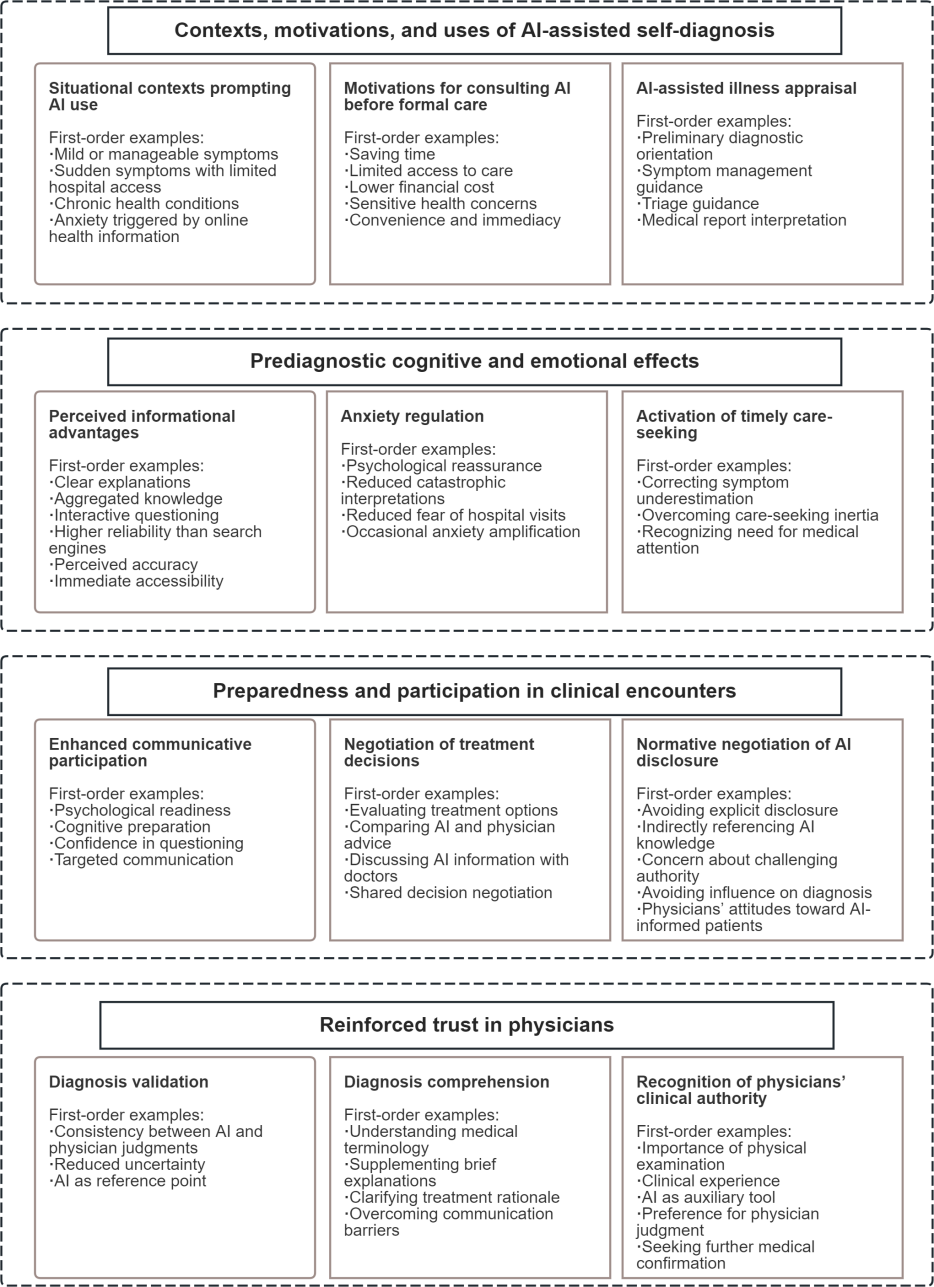
Thematic structure. AI: artificial intelligence.

Thematic analysis of interview data found 4 interrelated themes about how generative AI–assisted self-diagnosis is integrated into patients’ care-seeking processes. These themes correspond to the 4 qualitative research questions: contexts and motivations for AI use, pre-diagnostic effects of AI-assisted symptom interpretation, AI’s influence on communication during clinical visits, and the relationship between AI use and perceptions of professional authority. The coding system is shown in Figure 1.

### Drivers, Functions, and Perceptions of AI-Assisted Self-Diagnosis

AI-based health consultations clustered in a prediagnostic gray zone: Symptoms were concerning but not clearly serious enough to require immediate care, or professional care was not readily accessible or convenient. In this space, AI offered preliminary orientation without requiring users to commit to clinical action.

Common triggers included mild but persistent symptoms, such as skin irritation, gastrointestinal discomfort, or low-grade fever, and sudden symptoms arising at inconvenient times, especially at night or when alone. As Participant 9 put it, symptoms could be “not serious enough to go to the hospital...but uncomfortable if left untreated.”

AI use was also shaped by practical constraints, including time pressure, difficulty taking leave from work, long waits, complex hospital procedures, geographic distance, cost concerns, and embarrassment about sensitive symptoms. These constraints made AI attractive as a low-barrier first step rather than as a replacement for care.

Within these constraints, participants used AI for preliminary assessment; urgency judgment; department selection and interim self-management, such as wound care, pain relief strategies, or over-the-counter medication advice.

### Prediagnostic Outcomes of AI Use

Participants often contrasted AI-generated explanations with conventional internet searches. In their accounts, this contrast appeared to have a calibrating effect, helping them develop more proportionate interpretations of their symptoms and care needs.

For participants with a tendency toward health anxiety, AI use often mitigated excessive worry previously amplified by internet-based information-seeking. Several participants contrasted AI with traditional online searches, which they described as fragmentary and fear-inducing, frequently escalating minor symptoms into catastrophic possibilities. In comparison, AI was perceived as offering more structured and proportionate explanations of potential causes and risks. As Participant 22 noted, “After asking AI, I felt calmer. It didn’t exaggerate the risks, and I stopped imagining the worst.” In this sense, AI helped restrain overreaction by reducing unnecessary panic and interrupting cycles of reassurance-seeking.

Conversely, for participants who tended to delay or avoid medical care, AI helped them reassess the seriousness of their symptoms and consider seeking treatment. Individuals who described themselves as “afraid of trouble,” “too busy,” or generally reluctant to visit hospitals reported that, when AI indicated that a condition might require professional evaluation, they were more likely to overcome inertia and take action. As Participant 1 explained, “AI said this problem shouldn’t be delayed and should be treated in a major hospital. That made me stop thinking it would just go away on its own.” Another participant similarly noted that AI sometimes prompted more proactive self-care: “Before, I might not even take medicine. But after asking AI, I would at least go buy some medicine” (Participant 21).

For others, AI also reduced uncertainty about navigating the health care system. As Participant 16 explained, consulting AI beforehand helped clarify which department to visit and what examinations might be needed, thereby lowering the anxiety associated with hospital visits.

Together, these pathways show why we describe AI-facilitated symptom interpretation as calibrated illness appraisal: AI could dampen unwarranted anxiety while countering avoidance when professional care appeared necessary.

### Preparedness and Participation in Clinical Encounters

Participants consistently reported that consulting generative AI before medical visits improved their understanding of health concerns and confidence in communicating with physicians. AI helped them become familiar with medical terminology, possible explanations for symptoms, and treatment options, enabling them to enter consultations with clearer priorities and more focused questions. This preparation supported a shift from passive receipt of medical advice toward more active dialogue. As Participant 42 reflected, “I already have a rough idea about my condition, and I’m no longer afraid to ask questions—I don’t just sit there waiting for the doctor to decide everything for me.” Participant 25 similarly noted that, whereas questions previously arose only after leaving the hospital, AI-assisted preparation enabled them to “bring a list of focused questions and get them answered directly.”

An illustrative case involved recurrent periodontitis (Participant 16). After experiencing a sudden flare-up of tooth pain late at night and being unable to seek immediate care, the participant consulted AI. The AI suggested that irrigation might provide symptom relief and could be considered as an alternative to medication or extraction. At a dental visit the following day, the dentist initially recommended extraction. Because the participant feared extraction and had learned that irrigation might be an option, they asked whether it could be attempted first. The dentist agreed that irrigation could provide relief and proceeded with that approach.

This episode illustrates how AI-assisted self-diagnosis may facilitate negotiated decision-making by reducing informational barriers to preference expression. A clinician may initially recommend a standard treatment without fully knowing the patient’s concerns, while a patient who is unaware of alternatives may be unable to articulate an actionable preference. In this case, AI made irrigation visible as a possible option and provided the participant with the language to raise it. This enabled the dentist to consider the patient’s fear of extraction alongside clinical judgment, supporting a more individualized and acceptable treatment plan.

However, this participation was often discreet. Many participants concealed that their questions or suggestions were AI-informed, fearing that disclosure might signal mistrust or challenge professional authority. Instead, they reframed AI-derived insights as personal reflections or tentative inquiries. As Participant 12 explained, “I didn’t tell the doctor I had used AI; I was worried he’d think I didn’t trust him and then treat me differently.” Although AI shaped participants’ preparation, reasoning, and communication, its role was often hidden because of anticipated authority norms. By contrast, those who disclosed AI use generally reported constructive physician responses, including fuller explanations and more patient-centered dialogue. This suggests that perceived social expectations, rather than actual professional resistance, may constrain the communicative potential of AI-assisted preparation.

### Trust Reinforcement Through Diagnosis Validation and Comprehension

Most participants reported that AI reinforced, or at least maintained, trust in physicians through 2 processes: diagnosis validation and diagnosis comprehension.

Diagnosis validation occurred when AI-generated assessments aligned with physicians’ clinical judgments. Participants frequently described AI and physicians as “pointing in the same direction,” a convergence that strengthened confidence in physicians’ diagnoses or treatment recommendations. For example, in a case involving persistent stomach pain, a participant initially suspected infection with a widely discussed virus strain (Participant 21). Both the AI and the physician suggested that the symptoms were more likely related to recent dietary habits than to a viral infection. Because the AI and the physician converged against the participant’s initial assumption, the participant became more willing to accept the physician’s diagnosis and reported greater trust in the doctor.

Diagnosis comprehension operated through a complementary mechanism. Rather than validating the correctness of the clinical outcome, AI helped participants understand physicians’ reasoning by translating brief or technical explanations into more accessible language. Participants noted that consultations were often time-constrained, leaving limited opportunity for detailed explanation. In this context, AI functioned as an explanatory supplement, enabling patients to reconstruct why a physician recommended a particular diagnosis, medication, test, or treatment plan. For some participants, this interpretive support reduced suspicion that physicians were overprescribing or recommending unnecessary treatments, thereby strengthening trust.

Negative and discrepant cases qualified this pattern. Minor differences usually involved terminology, emphasis, medication choice, self-management advice, cost, or treatment preference; participants typically used these differences to ask questions, seek clarification, or consult another physician rather than reject medical authority.

One substantial discordance involved shingles (Participant 48). AI suggested that radiating pain could fit shingles, whereas the first physician recommended an additional breast examination because no rash was visible. After another physician later confirmed that radiating pain could occur with shingles and no breast pathology was found, the participant reported reduced trust in the first physician.

This negative case shows that AI can make trust in an individual physician more conditional when substantial discordance is later validated. Still, it did not represent the replacement of physicians by AI: The participant continued seeking medical confirmation and treatment. The dominant pattern was therefore the reinforcement of trust, with discordance functioning as an important boundary condition.

### Survey Results

**Figure 2.**
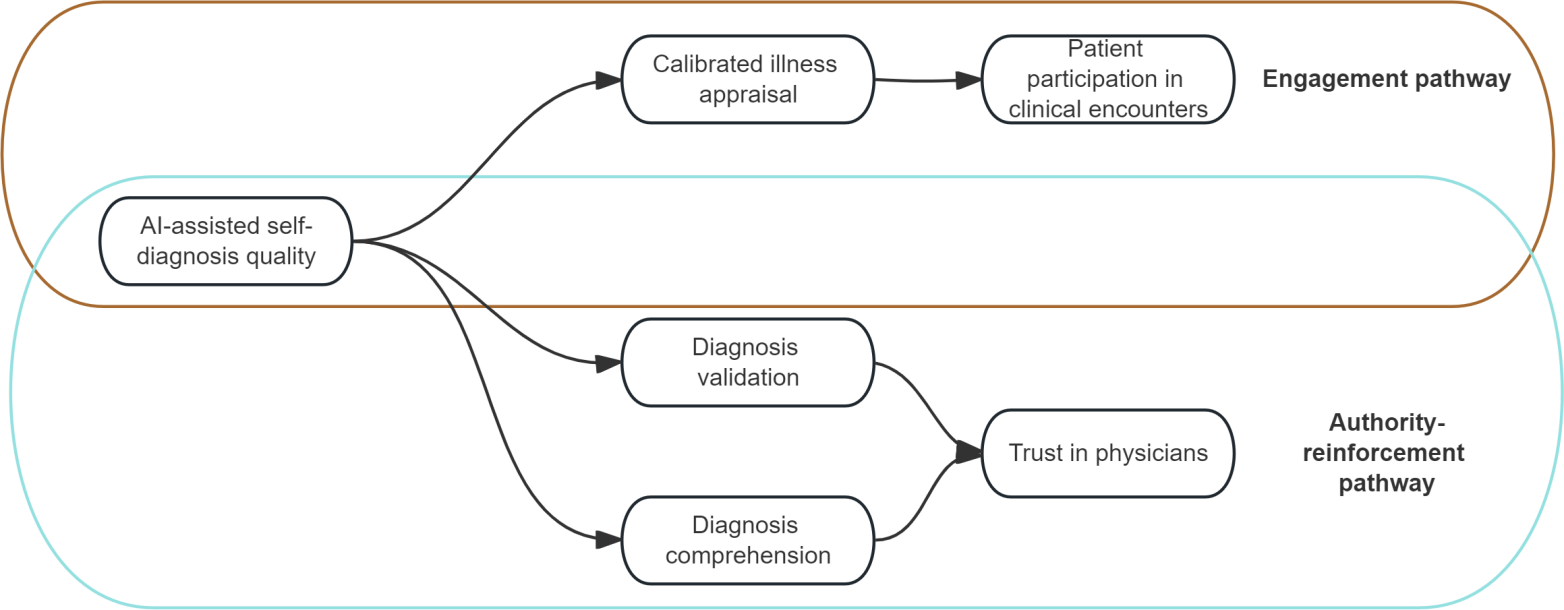
Conceptual framework. AI: artificial intelligence.

The quantitative study aimed to examine whether the 2 pathways identified in the qualitative phase could also be observed in the survey data (see Figure 2). The first pathway concerned illness appraisal calibration, encompassing the associations among perceived quality of AI-assisted symptom interpretation, more proportionate assessments of symptom severity, and patient participation in clinical encounters. The second pathway concerned authority reinforcement, encompassing the associations among AI-generated explanations, diagnosis validation, diagnosis comprehension, and trust in physicians.

### Descriptive Statistics and Correlations

Respondents reported varying levels of trust in different sources of health information, with each source assessed using a single item. Trust in physicians as a health information source (mean 3.82, SD 1.07) and government health agencies (mean 3.80, SD 1.10) were perceived as the most trustworthy sources. Relatively high trust was also reported for AI chatbots (mean 3.66, SD 1.02) and interpersonal sources such as family or friends (mean 3.63, SD 1.09). In contrast, traditional media and online platforms received lower trust ratings, including newspapers or magazines (mean 3.31, SD 1.05), television (mean 3.30, SD 1.05), search engines (mean 3.26, SD 1.11), radio (mean 3.22, SD 1.02), social media platforms (mean 3.11, SD 1.05), blogs (mean 3.08, SD 1.10), and online news platforms (mean 3.07, SD 1.13). Overall, trust in AI chatbots approached the level of interpersonal sources but remained lower than trust in physicians as a health information source and trust in government health agencies.

Table 3 summarizes the bivariate correlations among the key study variables. Mean scores for all focal constructs were above the scale midpoint, indicating generally favorable perceptions of AI-assisted self-diagnosis and its role in clinical encounters: perceived quality of AI-assisted self-diagnosis (mean 3.70, SD 0.86), calibrated illness appraisal (mean 3.79, SD 0.88), patient participation in clinical encounters (mean 3.75, SD 0.87), diagnosis validation (mean 3.66, SD 0.97), diagnosis comprehension (mean 3.72, SD 0.95), and trust in physicians (mean 3.57, SD 0.85). All key constructs were significantly and positively correlated, providing preliminary support for the proposed relationships among AI-assisted self-diagnosis, consultation processes, and trust in physicians.

**Table 3.** Correlations among the key study variables.

Variable	Perceived quality of AI-assisted self-diagnosis	Calibrated illness appraisal	Patient participation in clinical encounters	Diagnosis validation	Diagnosis comprehension	Trust in physicians
Perceived quality of AI-assisted self-diagnosis						
*r*	—^a^	0.55	0.61	0.61	0.59	0.49
*P* value	—	<.001	<.001	<.001	<.001	<.001
Calibrated illness appraisal						
*r*	0.55	—	0.58	0.53	0.59	0.47
*P* value	<.001	—	<.001	<.001	<.001	<.001
Patient participation in clinical encounters						
*r*	0.61	0.58	—	0.59	0.63	0.45
*P* value	<.001	<.001	—	<.001	<.001	<.001
Diagnosis validation						
*r*	0.61	0.53	0.59	—	0.55	0.44
*P* value	<.001	<.001	<.001	—	<.001	<.001
Diagnosis comprehension						
*r*	0.59	0.59	0.63	0.55	—	0.46
*P* value	<.001	<.001	<.001	<.001	—	<.001
Trust in physicians						
*r*	0.49	0.47	0.45	0.44	0.46	—
*P* value	<.001	<.001	<.001	<.001	<.001	—

a Not applicable.

### Engagement Pathway

The regression model (Table 4) predicting calibrated illness appraisal was significant (*F*_1,544_=238.88, *P*<.001; *R*²=0.3051). Perceived quality of AI-assisted self-diagnosis was positively associated with calibrated illness appraisal (*b*=0.57, 95% CI 0.49-0.64). The regression model predicting patient participation was also significant (*F*_2,543_=229.09, *P*<.001; *R*²=0.4576). Calibrated illness appraisal was positively associated with patient participation after accounting for perceived AI quality (*b*=0.35, 95% CI 0.28-0.43). The direct association between perceived AI quality and patient participation also remained significant (*b*=0.42, 95% CI 0.35-0.50).

Bootstrap analysis indicated a significant indirect association between perceived AI quality and patient participation through calibrated illness appraisal (indirect effect=0.20, 95% bootstrap CI 0.14-0.26). Thus, perceived AI quality was associated with patient participation both indirectly through calibrated illness appraisal and directly after the mediator was included. These findings supported H1‐H3.

**Table 4.** Mediation model of calibrated illness appraisal; all coefficients are unstandardized.

Path and effect	Estimate (95% CI)	SE	*t* (df)	*P* value
Path coefficients				
QLT^a^ → CIA^b^	0.57 (0.49-0.64)	0.04	15.46 (544)	<.001
QLT → PTCP^c^ (direct effect)	0.42 (0.35-0.50)	0.04	10.88 (543)	<.001
CIA → PTCP	0.35 (0.28-0.43)	0.04	9.35 (543)	<.001
Indirect effect (bootstrap)				
QLT → CIA → PTCP	0.20 (0.14-0.26^d^)	0.03^e^	N/A^f^	N/A

a QLT: perceived quality of artificial intelligence (AI)–assisted self-diagnosis.

b CIA: calibrated illness appraisal.

c PTCP: patient participation in clinical encounters.

d Bootstrap CI.

e Bootstrap SE.

f N/A: not applicable.

### Authority Reinforcement Pathways

The regression model (Table 5) predicting diagnosis validation was significant (*F*_1,544_=317.62, *P*<.001; *R*²=0.3686). Perceived quality of AI-assisted self-diagnosis was positively associated with diagnosis validation (*b*=0.69, 95% CI 0.61-0.76). The model predicting diagnosis comprehension was also significant (*F*_1,544_=291.90, *P*<.001; *R*²=0.3492). Perceived AI quality was positively associated with diagnosis comprehension (*b*=0.65, 95% CI 0.58-0.73). The regression model predicting trust in physicians was significant (*F*_3,542_=77.08, *P*<.001; *R*²=0.2990). After perceived AI quality and both mediators were included, diagnosis validation (*b*=0.15, 95% CI 0.07-0.23) and diagnosis comprehension (*b*=0.20, 95% CI 0.12-0.28) were both positively associated with trust in physicians. The direct association between perceived AI quality and trust in physicians also remained significant (*b*=0.25, 95% CI 0.15-0.34; SE 0.05).

Bootstrap analysis indicated a significant total indirect association between perceived AI quality and trust in physicians (total indirect association=0.23, 95% bootstrap CI 0.15-0.31). Both specific indirect associations were significant: The indirect association through diagnosis validation was 0.10 (95% bootstrap CI 0.04-0.17), and the indirect association through diagnosis comprehension was 0.13 (95% bootstrap CI 0.07-0.19). Thus, perceived AI quality was associated with trust in physicians indirectly through both diagnosis validation and diagnosis comprehension, while a significant direct association remained after the mediators were included. These findings were consistent with H4‐H8.

**Table 5.** Mediation model of authority reinforcement.

Path and effect	Estimate (95% CI)	SE	*t* (df)	*P* value
Path coefficients				
QLT^a^ → DV^b^	0.69 (0.61-0.76)	0.04	17.82 (544)	<.001
QLT → DC^c^	0.65 (0.58-0.73)	0.04	17.09 (544)	<.001
QLT → TiP^d^ (direct effect)	0.25 (0.15-0.34)	0.05	5.14 (542)	<.001
DV → TiP	0.15 (0.07-0.23)	0.04	3.55 (542)	<.001
DC → TiP	0.20 (0.12-0.28)	0.04	4.73 (542)	<.001
Indirect effects (bootstrap)				
Total indirect effect	0.23 (0.15-0.31^e^)	0.04^f^	N/A^g^	N/A
QLT → DV → TiP	0.10 (0.04-0.17^e^)	0.03^f^	N/A	N/A
QLT → DC → TiP	0.13 (0.07-0.19^e^)	0.03^f^	N/A	N/A

a QLT: perceived quality of artificial intelligence (AI)–assisted self-diagnosis.

b DV: diagnosis validation (perceived consistency between AI assessments and physicians’ diagnoses).

c DC: diagnosis comprehension (understanding physicians’ diagnostic reasoning through AI-assisted explanations).

d TiP=trust in physicians.

e Bootstrap CI.

f Bootstrap SE.

g N/A: not applicable.

### Integrated Mixed Methods Findings

Table 6 integrates the qualitative and quantitative findings. Survey results converged with interview themes: perceived AI quality was associated with calibrated illness appraisal and participation, while AI-generated explanations were associated with trust in physicians through diagnosis validation and comprehension. Overall, AI-assisted self-diagnosis functioned mainly as an intermediary rather than a substitute for physicians.

**Table 6.** Joint display of integrated findings.

Care-seeking phase or mechanism	Qualitative findings	Quantitative evidence	Integrated meta-inference
Prediagnostic orientation	Participants described using AI^a^ in a prediagnostic gray zone to interpret uncertain symptoms, regulate disproportionate anxiety, or reconsider seeking care when delay appeared risky.	Higher perceived AI quality was positively associated with calibrated illness appraisal.	Perceptions of clearer AI outputs were associated with more proportionate illness appraisal rather than simple reassurance or alarm.
Consultation participation	Participants perceived AI as helping them organize symptoms, learn terminology, and formulate questions, although many concealed its use from physicians.	Calibrated illness appraisal showed a significant indirect association between perceived AI quality and patient participation.	The findings were consistent with an association between AI-supported appraisal, preconsultation sense-making, and more active participation.
Diagnosis validation	Participants reported greater confidence in diagnoses or treatment recommendations when AI-generated assessments aligned with physicians’ judgments.	Higher perceived AI quality was associated with diagnosis validation, which was positively associated with trust in physicians.	Alignment between AI outputs and professional judgment was associated with stronger trust in physicians.
Diagnosis comprehension	Participants described AI as making brief or technical clinical explanations more accessible.	Higher perceived AI quality was associated with diagnosis comprehension, which was positively associated with trust in physicians.	Greater AI-assisted understanding of physicians’ reasoning was associated with stronger trust in physicians.
Boundary condition: discordance	In rare cases, trust in an individual physician became more conditional when AI advice was later supported by another clinician or subsequent outcomes.	Case-specific discordance was not directly examined in the survey; the quantitative findings were consistent with the overall trust-supportive pathways identified qualitatively.	AI use was generally compatible with continued reliance on medical authority, while validated discordance was sometimes accompanied by more conditional trust in an individual physician.

a AI: artificial intelligence.

## Discussion

This study examined how generative AI-assisted self-diagnosis is incorporated into care-seeking trajectories and how it relates to patient participation and trust in physicians. Across the interview and survey findings, generative AI was usually described not as a substitute for physicians but as an informational intermediary. It helped users interpret symptoms before consultation, prepare for communication with clinicians, and make sense of diagnoses and treatment rationales after the encounter.

### AI-Assisted Preparedness and Patient Participation

The value of generative AI as a provisional interpretive resource becomes particularly evident in how patients prepare for and participate in clinical encounters. Research from the internet era indicates that preconsultation health information seeking can help patients clarify symptoms and reduce uncertainty [40,41]. However, fragmented, inconsistent, or alarmist information may also increase anxiety and confusion, limiting its preparatory and empowering potential [14].

In participants’ accounts, this ambivalence appeared less pronounced when using generative AI. Many described AI-generated explanations as helping them develop more proportionate emotional and behavioral responses to health concerns. For anxious users, clear and tailored explanations made symptoms more understandable and less overwhelming. For those inclined to delay care, generative AI could encourage timely and appropriate treatment-seeking by drawing attention to potential risks and emphasizing the need for professional evaluation when warranted.

This sense-making also shaped communication during consultations. Participants reported that AI helped them identify clinically relevant symptoms, organize their accounts, and formulate questions before meeting physicians. This finding aligns with research showing that meaningful patient participation depends not only on physicians’ willingness to communicate but also on patients’ ability to articulate concerns and interpret medical explanations [36,37,42]. AI-assisted self-diagnosis may therefore function as a preconsultation communication resource, helping patients enter clinical encounters better prepared to describe symptoms, ask questions, and engage in dialogue without replacing professional judgment.

However, greater preparedness did not necessarily lead to open disclosure of AI use. Many participants incorporated AI-generated information into their questions or symptom narratives without telling physicians where the information originated. This pattern echoes findings from the internet era, when patients sometimes concealed online information because they feared challenging physicians’ authority or consuming limited consultation time [43,44]. However, participants who openly discussed AI-generated information generally described physicians’ responses as constructive and reported receiving more detailed explanations. This contrast suggests that patients may anticipate more resistance than physicians actually display. More open discussion of AI-assisted information seeking may therefore improve communication transparency and support collaborative dialogue in clinical encounters.

### AI-Mediated Trust in Health Care

This study found that respondents who perceived AI-generated assessments as consistent with physicians’ judgments and who reported that AI helped them understand clinical reasoning also tended to report greater trust in physicians. In other words, AI-assisted self-diagnosis was generally associated with stronger, rather than weaker, trust in physicians through 2 related mechanisms: diagnosis validation and diagnosis comprehension.

Diagnosis validation arose when AI-generated assessments converged with physicians’ judgments. Participants often treated such agreement as independent confirmation of a diagnosis or treatment recommendation, making medical conclusions easier to accept. This finding challenges the assumption that algorithmic systems necessarily undermine professional authority or shift epistemic control away from physicians [45-47].

Responses to disagreement further qualify this pattern. Although previous studies suggest that conflicting recommendations may weaken trust in either AI or clinicians [2,26-28,48], discrepancies in this study were uncommon and rarely led participants to favor AI. Most instead sought clarification, interpreted AI outputs in light of clinical explanations, or consulted another physician, indicating that professional judgment remained primary, consistent with prior evidence of greater trust in human physicians than AI [49,50]. Nevertheless, when AI advice was later supported by another clinician or by subsequent outcomes, trust in a particular physician could become more conditional. AI may therefore reinforce confidence in medical expertise while enabling patients to scrutinize individual clinical decisions.

Diagnosis comprehension concerned AI’s role in making clinical reasoning more intelligible. Brief consultations, informational asymmetries, and limited explanations can leave patients uncertain about diagnoses and treatment choices [42,51-53]. Participants used AI to clarify technical information about tests, medications, diagnoses, and treatment rationales. In doing so, AI reduced perceived opacity and helped patients engage more meaningfully in clinical conversations.

Trust-related concerns appeared to arise less from the involvement of AI as a secondary source of knowledge than from physicians’ communicative responses, particularly when patients’ questions were dismissed or explanations were withheld. This is consistent with evidence that inadequate explanation and dismissive communication can damage patient-physician relationships [7].

Taken together, these findings suggest that generative AI can function as an informational intermediary in health care. It may support trust by confirming clinical judgments and by making the reasoning behind them more understandable. In this sample, AI-assisted self-diagnosis generally supplemented rather than displaced medical authority.

### Generative AI as a Symptom Interpretation Resource

This study found that generative AI functioned primarily as a provisional symptom interpretation resource rather than an autonomous diagnostic authority or substitute for physicians. Participants typically used AI under conditions of uncertainty, when symptoms had emerged but professional care did not yet seem necessary or was not immediately available or convenient. In these situations, AI helped users interpret symptoms, assess urgency, and prepare for possible medical consultation. Its perceived usefulness therefore lay in supporting illness appraisal, informal triage, and consultation preparation rather than delivering definitive diagnoses.

This finding complements research evaluating AI-assisted self-diagnosis from a clinical accuracy perspective. Previous studies have examined whether generative AI can produce accurate diagnoses or reliable triage recommendations [16,54,55]. Although its diagnostic and triage performance remains inconsistent, making it unsuitable as a standalone clinical tool [16], our findings suggest that it may still be useful within a provisional, user-directed, self-diagnosis process.

The difference reflects distinct analytic focuses. Accuracy-oriented studies assess whether AI outputs meet expert clinical standards, whereas this study examined how users incorporate them into real-world care-seeking trajectories. Users may compare AI not with physicians as an equivalent authority but with conventional internet searches, personal experience, or making decisions with limited information. Consistent with research on online health information-seeking, digital resources may be valued for immediacy, convenience, and accessibility as well as authority [56-58]. Generative AI may therefore offer a relative informational advantage under uncertainty and constrained access to professional advice without being regarded as clinically equivalent to physicians.

This interpretation aligns with the human-in-the-loop model of technology-supported, self-triage decision-making by Kopka et al [17]. In this model, technology supports information-gathering and preliminary analysis, while users integrate its outputs with bodily experiences, prior knowledge, emotional responses, practical constraints, and professional advice. Similarly, participants interpreted AI-generated explanations in relation to their symptoms and circumstances rather than accepting them as final decisions.

This study extends this model by locating AI-assisted self-triage within a broader care-seeking trajectory. AI use often began in a prediagnostic gray zone before users had decided whether formal care was warranted and supported both early illness appraisal and explicit triage decisions. Its influence also extended into clinical encounters and postconsultation interpretation, shaping patient participation, diagnosis validation, and diagnosis comprehension. Technology-supported self-triage may therefore be understood as a longer process connecting symptom interpretation, medical consultation, and trust in physicians.

### Limitations and Implications

Several limitations should be noted. Both phases were conducted in China, and purposive qualitative sampling and convenience survey sampling limit generalizability. Participants who had used AI for health consultation and agreed to participate may have been more favorable toward AI or more comfortable discussing health information seeking than nonparticipants. The study relied on retrospective self-reports rather than observation of clinical encounters, and the cross-sectional survey cannot establish temporal order or causality. The mediation models were unadjusted for potential confounders, so the indirect pathways should be interpreted as theoretically specified associations rather than causal effects. Finally, although coding decisions were reviewed within the research team, the first author’s primary role in coding may have shaped theme development.

The findings nevertheless suggest practical implications. Patient guidance should frame generative AI as a resource for symptom organization, question preparation, and comprehension of medical information while warning against using AI as a definitive diagnostic authority. Clinicians may benefit from inviting patients to discuss AI-generated information so that misunderstandings can be corrected and clinical reasoning can be clarified. AI system design should communicate uncertainty, encourage professional evaluation when appropriate, and support consultation preparation rather than simulate diagnostic certainty.

Future research should examine when AI functions as a complementary informational resource and when it becomes a competing authority. Longitudinal studies, experiments, and direct observation of consultations are needed to test temporal ordering among AI use, calibrated appraisal, communication behaviors, and trust in physicians. Future work should also validate calibrated illness appraisal, diagnosis validation, and diagnosis comprehension as mechanisms linking AI-assisted self-diagnosis with patient participation and trust in physicians across health systems and clinical contexts.

### Conclusion

By synthesizing qualitative accounts and cross-sectional survey data, this study provided mixed methods evidence on how generative AI-assisted self-diagnosis is integrated into patients’ care-seeking trajectories and clinical encounters. Participants generally did not perceive generative AI as a competing source of medical authority; instead, they used it as an informational intermediary to interpret symptoms, prepare for clinical consultations, and contextualize physicians’ diagnoses and clinical reasoning. These findings suggest that generative AI–assisted self-diagnosis can be compatible with patient-physician relationships when it supports patients’ interpretive and communicative preparation rather than replacing professional judgment.

## Supplementary material

10.2196/95072 Multimedia Appendix 1 Interview questions and survey items.

## Abbreviations

AI: artificial intelligence
CFI: comparative fit index
H: hypothesis
LLM: large language model
RMSEA: root mean square error of approximation
RQ: research question
SRMR: standardized root mean squared residual
TLI: Tucker-Lewis index
