# Supplementary material for: Generative Artificial Intelligence (AI)–Assisted Self-Diagnosis and the Patient-Physician Relationship: Mixed Methods Study of Calibration, Participation, and Trust
Source: J Med Internet Res. 2026 Aug 18;28:e95072. doi: 10.2196/95072 (PMC13484928; doi:10.2196/95072)
Supplement: Supplementary file 1 [file jmir-v28-e95072-s001.docx]

| **Table S1. Outline of interview questions.** | |
| --- | --- |
| No. | Questions |
| 1 | Could you tell me about any experiences you’ve had using artificial intelligence to seek health information or advice? If so, please describe your most recent or most memorable experience using an AI tool for this purpose. |
| 2 | After consulting the AI, what do you usually do next? |
| 3 | When you later visit a doctor, do you mention that you’ve already checked your symptoms or received advice from an AI? How do doctors typically respond, and how do you feel about their reactions? If you usually don’t tell them, could you share why? |
| 4 | In what ways, if any, has your experience of seeking medical care changed since you began using AI? |
| 5 | Has using AI affected your trust in doctors in any way? Could you give an example or explain why? |
| 6 | If the AI’s suggestions differ from what the doctor says, how do you usually handle that situation? |
| 7 | How reliable do you think medical AI is? Do you personally trust it? Why or why not? |
| 8 | How familiar are you with medical AI and how it works? |
| 9 | To what extent do you think medical AI can play a role in healthcare decision-making? |
| 10 | What kinds of support or assistance would you like medical AI to provide during the healthcare process? |
| 11 | What role do you think doctors should play when using medical AI in their practice? |
| 12 | What are your hopes or expectations for the future development of medical AI? |
| 13 | Would you recommend using medical AI to others? Why or why not? |
| 14 | Is there anything else related to using AI for health inquiries or interacting with doctors that we haven’t talked about but you think is important or would like to add? Are there any additional thoughts that came to mind during the interview that you haven’t yet mentioned? |

**Table S2. Survey items used for the main analytic variables.**

| Domain | Item | Item wording (English translation) |
| --- | --- | --- |
| Perceived quality of AI-assisted self-diagnosis | QLT1 | The health-related information provided by AI was generally easy to understand. |
|  | QLT2 | The information provided by AI was clear to read and understand. |
|  | QLT3 | AI was able to provide relatively comprehensive health-related information. |
|  | QLT4 | The information provided by AI was highly relevant to my current health problem. |
|  | QLT5 | The explanations or suggestions provided by AI closely matched my personal situation. |
|  | QLT6 | The health information provided by AI seemed accurate to me. |
|  | QLT7 | The health information provided by AI was generally trustworthy. |
| Calibrated illness appraisal | CIA1 | AI helped me judge the severity of my symptoms more reasonably. |
|  | CIA2 | By consulting AI, I gained a clearer understanding of possible causes of my current health problem. |
|  | CIA3 | After consulting AI about a health problem, I no longer felt overly anxious about some health conditions that were not serious. |
|  | CIA4 | When facing a health problem, the information from AI helped me view my condition more calmly rather than magnifying my worries. |
|  | CIA5 | After consulting AI about a health problem, I was less likely to ignore situations requiring medical care and would seek professional help in a timely way. |
| Patient participation in clinical encounters | PTCP1 | AI helped me ask doctors questions more actively during medical consultations. |
|  | PTCP2 | AI helped me participate more deeply in discussions about treatment plans. |
|  | PTCP3 | AI made it easier for me to express my own views or opinions during diagnosis and treatment. |
|  | PTCP4 | AI helped me make fuller use of consultation time with the doctor. |
| Diagnosis validation | DV1 | The diagnosis given by the doctor was generally consistent with the diagnosis suggested by AI. |
|  | DV2 | The advice given by the doctor was generally consistent with AI's judgment. |
|  | DV3 | AI and the doctor mutually confirmed each other's views about my health problem. |
| Diagnosis comprehension | DC1 | AI helped me more clearly understand the reasons behind the doctor's diagnosis or treatment decision. |
|  | DC2 | With the help of AI, it was easier for me to understand why the doctor chose the current treatment plan rather than other possible options. |
|  | DC3 | AI partly compensated for aspects of the consultation that I did not fully understand. |
| Trust in physicians | TMP1 | Sometimes doctors care more about what is convenient for them than about their patients' medical needs. |
|  | TMP2 | Doctors are extremely thorough and careful. |
|  | TMP3 | I completely trust doctors’ decisions about which medical treatments are best. |
|  | TMP4 | A doctor would never mislead patients about anything. |
|  | TMP5 | All in all, I trust doctors completely. |
| Trust in health information sources | HIT1 | How much do you trust health or medical information from doctors? |
|  | HIT2 | How much do you trust health or medical information from family or friends? |
|  | HIT3 | How much do you trust health or medical information from newspapers or magazines? |
|  | HIT4 | How much do you trust health or medical information from radio? |
|  | HIT5 | How much do you trust health or medical information from online news? |
|  | HIT6 | How much do you trust health or medical information from television? |
|  | HIT7 | How much do you trust health or medical information from government health institutions? |
|  | HIT8 | How much do you trust health or medical information from social media? |
|  | HIT9 | How much do you trust health or medical information from search engines? |
|  | HIT10 | How much do you trust health or medical information from blogs? |
|  | HIT11 | How much do you trust health or medical information from large language models such as ChatGPT, DeepSeek, or Doubao? |

Note: Items were administered in Chinese; the wording above is an English translation for reporting.
